# Effect of Cluster Set Configurations on Mechanical Variables During the Deadlift Exercise

**DOI:** 10.2478/hukin-2013-0064

**Published:** 2013-12-31

**Authors:** Gavin L. Moir, Bruce W. Graham, Shala E. Davis, John J. Guers, Chad A. Witmer

**Affiliations:** 1 Exercise Science Department, East Stroudsburg University. USA.

**Keywords:** interrepetition rest, fatigue, resistance exercise

## Abstract

The purpose of the present study was to investigate the effects of different configurations of repetitions within a set of deadlifts on the mechanical variables of concentric force, concentric time under tension, impulse, work, power, and fatigue. Eleven resistance trained men (age: 21.9 ± 1.0 years; deadlift 1 repetition maximum: 183.2 ± 38.3 kg) performed four repetitions of the deadlift exercise with a load equivalent to 90% of 1 repetition maximum under three different set configurations: Traditional (continuous repetitions); Doubles cluster (repetitions 1 and 2, and 3 and 4 performed continuously with a 30 s rest inserted between repetitions 2 and 3); Singles cluster (30 s rest provided between repetitions). The order of the sessions was counterbalanced across the subjects and the mechanical variables were calculated during each repetition from the synchronized signals recorded from force platforms and a motion analysis system. Relative to the Traditional set, the insertion of rest periods in the cluster set configurations resulted in greater time under tension (p < 0.001) and therefore, greater impulse (p < 0.001) during the repetitions. Reductions in power were observed during the cluster sets compared to the Traditional set (p = 0.001). The Doubles cluster set resulted in greater fatigue scores for power compared to the Traditional set (p = 0.04). The influence of cluster sets on mechanical variables appears to be mediated by the mechanical characteristics of the exercise (i.e. stretch-shortening cycle) and the competing physiological mechanisms of fatigue and potentiation.

## Introduction

Resistance training has long been advocated in the training of athletes as an effective method to improve muscular strength and power (Kraemer et al., 2002). Considerable research exists highlighting the many phenotypic and neurogenic factors that are proposed to underpin improvements in strength and power following a period of resistance training (Abernethy et al., 1994; Baldwin and Haddad, 2001; Duchateau et al., 2006; Folland and Williams, 2007). In order to accrue the appropriate adaptations required to improve athletic performance, it is necessary to manipulate the volume, intensity, and selection of resistance exercises, and these manipulations form the basis of developing a periodized training program (Bompa and Haff, 2008; Stone et al., 2007). One of the key elements to periodization is the management of fatigue that accumulates from repeated workouts (Plisk and Stone, 2003).

As the planned manipulation of training volume (number of repetitions, sets, workouts), intensity (load used) and exercise selection during a periodized resistance training program is implemented across different training timescales (macro-, meso-, microcycles, individual workouts), methods to manage fatigue vary depending upon the specific timescale (Stone et al., 2007). Cluster sets, whereby repetitions are interspersed with short rest periods (administered between consecutive repetitions [singles] or between groups of repetitions within the set [e.g. doubles or triples]) have been proposed as a method to manage fatigue during individual resistance training workouts by facilitating partial restoration of the metabolic and excitatory cellular environment (Haff et al., 2008). Typically, resistance training exercises are completed in a continuous manner during each set performed in a workout, leading to the onset of fatigue (Haff et al., 2003; Lawton et al., 2006; Rooney et al., 1994). While the evidence for the beneficial influence of accumulated fatigue during resistance training contributing to gains in muscular strength is conflicting (Folland et al., 2002; Rooney et al., 1994), fatigue has been suggested to interfere with the development of muscular power output (Tidow, 1990). Indeed, the insertion of short inter-repetition rest periods (20 – 130 s) during cluster sets has been shown to ameliorate the decrease in bar velocity and power output between repetitions completed in sets of both clean pulls and the bench press exercise compared to a continuous repetitions scheme (Denton and Cronin, 2006; Haff et al., 2003; Lawton et al., 2006). Furthermore, the advantage of short inter-repetition rest periods in maintaining power output during the bench press exercise appears to be unrelated to the specific configuration used, with schemes involving singles and doubles being equally effective (Lawton et al., 2006).

Most of the studies investigating the use of cluster sets with resistance exercises have focused on the mechanical variables of bar velocity and power output (Denton and Cronin, 2006; Haff et al., 2003; Lawton et al., 2006). These variables do not provide sufficient information for the strength and conditioning practitioner to determine the potential efficacy of cluster sets given the potential mechanical stimuli (e.g. force, impulse, work, power output) that are proposed to be important in the neuromuscular adaptations accrued from a period of resistance training (Crewther et al., 2005). Despite recent researchers including other mechanical variables (e.g. impulse, work (Denton and Cronin, 2006)), all of these studies are limited by the use of technologies that preclude the measurement of the ground reaction force during the movement, bringing the validity of the data into question (Cormie et al., 2007; Crewther et al., 2011). Therefore, the purpose of the present study was to investigate the effects of different configurations of repetitions within a set of deadlifts on the mechanical variables of force, impulse, work, power, concentric time and fatigue recorded using force platforms and a motion analysis system.

## Material and Methods

The study employed a crossover design in which subjects were required to perform four repetitions of the deadlift exercise with a load equivalent to 90% of 1-repetition maximum (1RM) under three different set configurations: Traditional set, where the repetitions were performed continuously; Doubles cluster set, where repetitions 1 and 2, and 3 and 4 were performed continuously with a 30 s rest inserted between repetitions 2 and 3; Singles cluster set, where 30 s rest was provided between each consecutive repetition. Each set configuration was performed during a separate testing session separated by a minimum of 72 hours and the order in which the sessions were administered was counterbalanced across the subjects.

### Participants

Eleven men agreed to participate in the present study (age: 21.9 ± 1.0 years; body height: 1.82 ± 0.08 m; body mass: 94.1 ± 19.4 kg; deadlift 1-RM: 183.2 ± 38.3 kg). All subjects had been free from any musculoskeletal injuries for the six months prior to testing and all had at least one year of resistance training experience. The study was approved by the Institutional Review Board at East Stroudsburg University and all subjects signed an informed consent form prior to testing.

### Procedures

Each subject attended four testing sessions across a two-week period, beginning with a familiarization and 1-RM testing session and followed by three sessions where the configuration of four repetitions of the deadlift performed with a load equivalent to 90% 1-RM was manipulated (Traditional set, Doubles cluster set [repetitions 1 and 2, and 3 and 4 are performed continuously with a 30 s rest inserted between repetitions 2 and 3], Singles cluster set [30 s rest between each consecutive repetition]). A minimum period of three days was allowed between the 1-RM test and the first experimental session, with a minimum period of 72 hours between the experimental sessions. Each subject completed their testing at the same time of day, and an Olympic barbell and bumper plates were used during all testing sessions.

### Measures

#### 1-RM testing

The 1-RM testing protocol of Baechle and Earle (2008) was used to assess the maximum deadlift load. Prior to testing, all subjects performed a standardized warm-up consisting of dynamic stretches. Subjects were instructed to keep the feet flat at all times during each repetition of the deadlift and to keep the back flat or slightly arched. The completion of the lift required the hips to be fully locked out. All subjects were allowed to use weightlifting chalk and a lifting belt. The 1-RM load provided the basis for all loads used for the experimental testing sessions.

#### Traditional set

During the Traditional set configuration, four repetitions of the deadlift exercise were performed continuously with a load equivalent to 90% 1-RM. Subjects were instructed to initiate the concentric phase of each repetition as soon as the bumper plates made contact with the ground, and to avoid bouncing the barbell on the ground between repetitions. This configuration prevented any rest period during the completion of the four repetitions. Prior to the experimental lifts, each subject completed a warm-up comprised of 5–7 minutes of dynamic exercises (10 walking lunges, 5 leg swings with each leg, and 10 shoulder/arm rolls) followed by four warm-up sets of the deadlift exercise (10 repetitions with a load equivalent to 50%, five repetitions with a load equivalent to 70% of 1-RM, three repetitions with a load equivalent to 80% 1RM, two repetitions with a load equivalent to 85% 1-RM). A 2–4 min rest period was given in between each set depending upon the load used.

#### Doubles cluster set

The first two repetitions were performed continuously, as per the Traditional set configuration. However, between repetitions two and three a 30 s rest period was inserted before repetitions three and four were completed in a continuous manner. The rest period was timed by the researchers and the subject was provided with a 10 s countdown to ensure that the third repetition was initiated at 30 s. Subjects were permitted to step away from the barbell during the rest period but remained standing. Each subject completed the same warm-up as that used during the Traditional set configuration.

#### Singles cluster set

A 30 s rest period was inserted between each repetition during this set configuration. The rest period was timed by the researchers and the subject was provided with a 10 s countdown to ensure that each repetition was initiated at 30 s. Subjects were permitted to step away from the barbell during the rest period but remained standing. Each subject completed the same warm-up as that used during the Traditional set configuration.

### Calculation of mechanical variables

The deadlifts during the three experimental sessions were performed with the subject standing on two force platforms (Kistler Type 9286AA) which were synchronized with a seven camera 3-dimensional (3D) system (Vicon, Oxford UK). The force platforms and 3D system sampled each repetition at a frequency of 200 Hz. A retro-reflective marker was placed in the center of the barbell to allow the vertical position of the barbell to be calculated. A repetition was defined as the event between the barbell being lifted from the ground to the barbell returning to the starting position, and was identified from the vertical acceleration of the retro-reflective barbell marker which was calculated using the second central difference method (Robertson et al., 2004). All mechanical variables were calculated during the ascent of the barbell, from the starting position to the highest vertical displacement during the repetition, which was defined as the concentric phase of the movement.

Both the vertical position of the barbell marker and the force data were filtered (generalized cross-validated quintic spline procedure) prior to the calculation of the following mechanical variables:

#### Average concentric force

The mean vertical ground reaction force during the concentric phase was calculated from the start of each repetition to the highest vertical position of the barbell to provide average concentric force (F). The force value was normalized to the subject’s body mass using the allometric parameter of ⅔ (Jaric, 2002). The reliability of this variable was assessed by calculating the intraclass correlation coefficient (ICC) from the data collected during the Traditional set configuration and was found to be 0.89, while the coefficient of variation (CV) was 4.1%.

#### Concentric time under tension

The time between the start of a repetition to the highest vertical position of the barbell was defined as the concentric time under tension (TUT) during each repetition. The ICC for this variable was 0.80 and the CV was 12.6%.

#### Impulse

The impulse of the vertical ground reaction force during the concentric phase of each repetition was calculated by integrating the force using trapezoidal integration (Robertson et al., 2004). The impulse was then normalized to each subject’s body mass using the allometric parameter of ⅔ (Jaric, 2002). The ICC for this variable was 0.87, with a CV of 13.0%.

#### Concentric work

The instantaneous mechanical work performed on the barbell during the concentric phase of each repetition was calculated by integrating the instantaneous power output using trapezoidal integration (Robertson et al., 2004). Concentric work was normalized to body mass using the allometric parameter of ⅔ (Jaric, 2002). This variable had an ICC of 0.83 and a CV of 6.1%.

#### Average concentric power output

The mean rate at which mechanical work was performed on the barbell during the concentric phase of each repetition was calculated as the product of the instantaneous vertical ground reaction force and the vertical velocity of the barbell. The instantaneous vertical velocity of the barbell was calculated from the vertical position of the retro-reflective marker placed on the barbell using the first central difference method (Robertson et al., 2004). The instantaneous power output was then averaged during each repetition to provide the average concentric power output (PO) and was normalized to body mass using the allometric parameter of ⅔ (Jaric, 2002). The ICC for this variable was 0.88, with a CV of 7.9%.

The fatigue score for the mechanical variables of average concentric force, impulse, average concentric work and average concentric power output during the different set configurations was calculated using the following equation (Fitzsimmons et al., 1993):

$$\begin{array}{l} \%\;\mathrm{Fatigue}=(100\times (\mathrm{total}\;\mathrm{mechanical}\;\mathrm{variable}\;\mathrm{value}÷\mathrm{ideal}\;\mathrm{mechanical}\;\mathrm{variable}\;\mathrm{value}))-100 \end{array}$$

where, total mechanical variable value = sum of the values from all repetitions, and ideal mechanical variable value = number of repetitions × greatest value across all repetitions within the set configuration.

### Statistical Analysis

All statistical analyses were performed using the Statistical Package for the Social Sciences (SPSS version 18.0). The normality of the data sets was confirmed (Shapiro-Wilk p > 0.05) and so central tendency and spread of the data were represented by means ± standard deviations. Differences in the mechanical variables during each repetition caused by the different set configurations were determined using an ANOVA model with repeated measures on two factors (set configuration: 3 levels; repetition: 4 levels). Simple contrasts were used to determine if the variables differed from the Traditional set and the first repetition. Differences in the percent fatigue scores were assessed using an ANOVA model with repeated measures on one factor (set configuration: 3 levels), with simple contrasts being used to determine if the values from the cluster sets differed from those in the Traditional set. The level of statistical significance for all analyses was set at p ≤ 0.05.

## Results

Table 1 shows the mechanical variables of average concentric force, impulse, concentric work, average concentric power output and concentric time under tension for the Traditional, Doubles cluster, and Singles cluster set configurations.

The ANOVA revealed a significant set configuration × repetition interaction for concentric TUT (p < 0.001) caused by the decrease in contraction time between repetitions 1 and 3 during the Traditional set being significantly different from the increase across these repetitions in the Doubles cluster set (p < 0.001). Similarly, the increase in contraction time between repetitions 1 and 2 (p = 0.006) and between repetitions 1 and 3 (p = 0.001) during the Singles cluster set was significantly different from the decrease observed in the Traditional set.

There was a significant set configuration × repetition interaction for impulse (p < 0.001). Simple contrasts showed that the decrease in the impulse between repetitions 1 to 3 in the Traditional set was significantly different from the increase in the impulse achieved between these repetitions in the Doubles cluster set (p = 0.002). Furthermore, the decrease in the impulse recorded between the first two repetitions in the Traditional set was significantly different from the increase between these two repetitions in the Singles cluster set (p = 0.018). Finally, the decrease between repetitions 1 and 3 in the Traditional set was significantly different from the increase recorded in the Singles cluster set between these repetitions (p = 0.007).

The ANOVA performed on the average concentric power output revealed a significant set configuration × repetition interaction (p = 0.001). This was caused by the decrease in power output between repetitions 1 to 2 (p = 0.010), 1 to 3 (p = 0.003) and 1 to 4 (p = 0.006) in the Singles cluster set configuration being significantly different from the increase observed across these repetitions during the Traditional set. Furthermore, the decrease in power output recorded between repetitions 1 and 3 in the Doubles cluster set configuration was significantly different from the increase recorded during the Traditional cluster set (p = 0.034).

The percent fatigue scores for each mechanical variable during the three set configurations are shown in Table 2.

There was a significant difference for the fatigue scores associated with power output (p = 0.04) caused by the greater fatigue associated with the Doubles set configuration compared to the Traditional set (p = 0.023).

## Discussion

The purpose of the present study was to investigate the effects of different configurations of repetitions within a set of deadlifts on the mechanical variables of force, impulse, work, power, time under tension and fatigue recorded using force platforms and a motion analysis system. The only mechanical variables that demonstrated positive effects as a result of the cluster sets in comparison to the Traditional set were those of concentric TUT and impulse with the increases in these variables observed across the cluster set configurations being greater than the reductions associated with the Traditional set. Given the lack of significant changes in average concentric force during these repetitions, the increased impulse was as a result of a greater time taken to perform the concentric phase of the movement. This increased concentric time was likely due to the absence of the stretch-shortening cycle (SSC) in the repetitions following the 30 s rest intervals inserted into the cluster set configurations. Specifically, a single repetition of the deadlift requires a concentric phase (barbell ascent) prior to an eccentric phase (barbell descent) and so the SSC may only be utilized when repetitions are performed continuously. Previous researchers have reported reduced concentric contraction times during resistance training movements involving the SSC compared to concentric-only exercises (Cronin et al., 2001; 2003; Newton et al., 1997). The Doubles cluster set configuration produced an increase in TUT between repetitions 1 and 3 while the Singles cluster set produced increases in TUT between repetitions 1 and 2 and 1 and 3 compared to the Traditional set. The affected repetitions occurred immediately after the 30 s rest and therefore the SSC was unlikely to be involved resulting in an increased concentric time. The time during which a muscle is under tension during a resistance training exercise is thought to be a potent stimulus of the hypertrophic response and gains in muscular strength following a period of resistance training (Crewther et al., 2005). The greater concentric contraction times observed in the present study during the cluster set configurations compared to the Traditional set combined with similar average concentric force values suggests that the use of repetitions separated by 30 s rest may present a greater stimulus to induce hypertrophy and strength gains using the deadlift exercise than performing the repetitions continuously. However, further research is required to substantiate this claim.

Although the effect of the cluster set configurations and the insertion of rest periods was largely positive for TUT and impulse, power output was negatively impacted. This finding is contrary to the findings of other researchers who have reported either increases in power output as a result of using cluster set configurations (Denton and Cronin, 2006; Lawton et al., 2006), or no change (Denton and Cronin, 2006; Haff et al., 2003). An explanation for the decreases in power output recorded in the present study is likely to relate once again to the absence of the SSC in the affected repetitions. Specifically, the 30 s rest interval between repetitions 2 and 3 in the Doubles cluster configuration precluded the involvement of a high-load eccentric contraction prior to the concentric phase of repetition 3, resulting in a power output that was reduced in comparison to that recorded during the Traditional set. The same mechanism is likely to be responsible for the reduced power outputs recorded during the final three repetitions during the Singles cluster set configuration. Interestingly, the power output during the final repetition of the Doubles cluster set configuration was greater than that achieved during the Traditional set. It is possible that the inclusion of a 30 s rest interval within the set, while precluding the use of the SSC during repetition 3 and the potentiating benefits associated with it (Komi, 2003), was sufficient to offset the deleterious effects of fatigue caused by continuous repetitions and allow for greater power output during repetition 4. Most previous studies investigating the effects of cluster sets on mechanical variables have used resistance training movements that involved large amplitude SSC, such as the bench press, and these studies tend to demonstrate improved power output (Denton and Cronin, 2006; Lawton et al., 2006). A single repetition of the bench press has an eccentric phase preceding the concentric phase and so the potentiating effect associated with the SSC can be expected.

Cluster sets have been proposed to facilitate the partial restoration of the cellular environment and therefore, reduce the accumulated fatigue that is associated with the completion of repetitions performed in a continuous manner (Haff et al., 2003; Lawton et al., 2006; Rooney et al., 1994). In the present study the Traditional set configuration produced fatigue for all the mechanical variables recorded, but the values were not statistically different from those in the Singles cluster set configuration; indeed, the Doubles cluster set actually produced significantly greater fatigue in power output compared to the Traditional set. These findings may reflect the competing mechanisms of concentric potentiation resulting from the utilization of the SSC (Komi, 2003) and neuromuscular fatigue during the concentric phases of the exercise. Specifically, the effects of fatigue that are manifest during the concentric phases of the deadlift during the Traditional set may be compensated for by the potentiating effect of the SSC; the absence of the SSC in some (Doubles cluster set) or all (Singles cluster set) of the repetitions performed in the cluster set configurations as a result of the deadlift exercise, which begins with a concentric phase, removes the potentiating effect of the SSC. However, the associated rest periods in the cluster configurations may allow the amelioration of fatigue. With these two competing mechanisms, the differences in fatigue between the Traditional set and the Singles cluster configuration are negligible in an exercise that does not utilize the SSC, such as a single repetition of the deadlift. The negative effect on fatigue recorded for average concentric power output during the Doubles cluster set configuration compared to the Traditional set may therefore be explained by the fact that the inclusion of an inter-repetition rest period and the utilization of the SSC during some of the repetitions (2 and 4) resulted in a greater variation in the mechanical variable, producing greater overall fatigue calculated using a percent decrement score. This highlights the need for strength and conditioning practitioners to consider the mechanics of the exercise, specifically the involvement of the SSC when administering interrepetition rest periods associated with cluster set configurations.

It has been proposed by some authors that fatigue is necessary to induce gains in muscular strength following a period of resistance training. Specifically, Rooney et al. (1994) reported that the gains in strength following a six week period of resistance training using repetitions performed continuously and which induced substantial fatigue, were greater than those observed following the training program where the training repetitions were performed as singles separated by a 30 s rest interval. The authors argued that the fatigue induced by the continuous repetition scheme would have required the involvement of high threshold motor units to complete the repetitions or the involvement of synergistic and antagonistic muscles, all of which would contribute to a greater expression of muscular strength following the training period. Contrary to these findings, Folland et al. (2002) reported that nine weeks of resistance training using a scheme to induce muscular fatigue during the workouts resulted in comparable increases in muscular strength compared to a scheme where fatigue was minimized. It is noteworthy that the low-fatigue workouts resulted in slightly greater overall training loads being used compared to the high-fatiguing workouts. Therefore, the role of fatigue in strength gains appears to be complex. In the present study, the effects of the different set configurations on fatigue, and therefore the potential influence on strength gains, were dependent upon the mechanical variable being measured. This complicates the influence of set configurations and how they may influence gains in muscular strength because the importance of the mechanical variables (e.g. force, impulse, work etc.) in contributing to the gains in strength observed following a period of resistance training is not yet fully understood (Crewther et al., 2005). It would therefore appear pertinent to identify the role of the specific mechanical variables associated with resistance training exercises in the gains of muscular strength before the efficacy of different set configurations can be determined.

A shortcoming of the present investigation is that the mechanical responses to the different configurations were studied following a single set. Exercises within a resistance training workout are typically performed across multiple sets and therefore investigating the mechanical responses to multiple sets will provide more practical information to the strength and conditioning practitioner. Intuitively one may not expect the pattern of mechanical responses observed during a single set to correspond to that following multiple sets given the interaction of the mechanisms that modulate contractile performance. Similarly, the pattern of responses observed here may not apply when the loads used during the exercise are manipulated such as during endurance, hypertrophy, and power schemes (Kraemer et al., 2002).

## Conclusion

The use of cluster set configurations would appear to confer benefits over the performance of continuous repetitions of the deadlift for the mechanical variable of impulse as a result of increased concentric TUT. This may mean that cluster sets involving the insertion of 30 s rest intervals between repetitions might provide a greater stimulus for strength and hypertrophy gains when using the deadlift exercise. However, given the negative effects on average concentric power output, the strength and conditioning practitioner should consider the interaction between the mechanics of the training exercise (e.g. involvement of the SSC) which is likely to influence the coexistence of fatigue and potentiation, the specifics of the set configuration (e.g. doubles, singles), as well as the importance of the mechanical variable (e.g. force, impulse, power output) in contributing to the desired adaptation when determining the potential efficacy of cluster sets during resistance training workouts.

## Figures and Tables

**Table 1:** The mechanical variables of average concentric force, impulse, concentric work, average concentric power output and concentric time under tension during the Traditional and Cluster set configurations. Values are means ± standard deviations

Set	Repetition	Mechanical variable				
		F (N/kg^⅔^)	Impulse (Ns/kg^⅔^)	Work (J/kg^⅔^)	PO (W/kg^⅔^)	TUT (s)
Traditional set	1	116 ± 16	235 ± 62^*†‡^	70.9 ± 9.1	33.5 ± 6.4^§**^	2.03 ± 0.48^*†‡^
	2	117 ± 14	202 ± 62	69.1 ± 8.1	38.8 ± 8.1	1.72 ± 0.48
	3	117 ± 12	202 ± 79	68.7 ± 10.9	39.5 ± 9.4	1.69 ± 0.53
	4	119 ± 13	244 ± 82	71.5 ± 9.6	35.3 ± 8.9	2.03 ± 0.64

Doubles cluster set	1	117 ± 23	228 ± 81^*^	71.8 ± 11.2	36.4 ± 7.9^**^	1.92 ± 0.48^*^
	2	120 ± 21	212 ± 76	71.9 ± 10.8	40.2 ± 8.8	1.74 ± 0.45
	3	113 ± 12	257 ± 76	71.0 ± 9.4	33.1 ± 8.4	2.29 ± 0.61
	4	120 ± 12	224 ± 75	70.3 ± 10.0	39.2 ± 8.1	1.83 ± 0.52

Singles cluster set	1	116 ± 12	229 ± 77^†‡^	70.7 ± 9.5	38.6 ± 11.4§	1.95 ± 0.54^†‡^
	2	121 ± 15	256 ± 90	72.5 ± 9.8	36.6 ± 8.7	2.08 ± 0.55
	3	118 ± 12	251 ± 83	71.8 ± 11.1	35.8 ± 7.7	2.09 ± 0.57
	4	117 ± 12	270 ± 76	71.5 ± 9.4	30.7 ± 8.0	2.30 ± 0.55

Doubles cluster set = repetitions 1 and 2, 3 and 4 performed continuously with a 30 s rest between repetitions 2 and 3; Singles cluster set = four repetitions performed with a 30 s rest between each consecutive repetition; Rep = repetition; F = average concentric force; PO = average concentric power output; TUT = concentric time under tension.

* Decrease between repetition 1 and 3 in Traditional set significantly different from increase in Doubles cluster set (p < 0.05)

† Decrease between repetition 1 and 2 in Traditional set significantly different from increase in Singles cluster set (p < 0.05)

‡ Decrease between repetition 1 and 3 in Traditional set significantly different from increase in Singles cluster set (p < 0.05)

§ Increase between repetition 1 and 2, 1 and 3, 1 and 4 in Traditional set significantly different from decrease in Singles cluster set (p < 0.05)

** Increase between repetitions 1 and 3 in Traditional set significantly different from decrease in Doubles cluster set (p < 0.05)

**Table 2:** Percent fatigue in the mechanical variables during the Traditional and cluster set configurations. Values are means ± standard deviations

Mechanical variable	Traditional set	Doubles cluster set	Singles cluster set
F	2.3 ± 2.8	5.2 ± 4.5	4.4 ± 4.6
Impulse	15.6 ± 9.9	14.0 ± 6.2	12.1 ± 5.6
Work	3.6 ± 3.8	4.9 ± 4.6	4.5 ± 3.7
PO	10.4 ± 5.3^*^	16.7 ± 6.0^*^	11.6 ± 6.9
TUT	16.4 ± 9.8	15.8 ± 7.7	11.8 ± 5.9

Doubles cluster set = repetitions 1 and 2, 3 and 4 performed continuously with a 30 s rest between repetitions 2 and 3; Singles cluster set = four repetitions performed with a 30 s rest between each consecutive repetition; F = average concentric force; PO = average concentric power output; TUT = concentric time under tension.

* Significantly different (p < 0.05)
